# Astrocytic engagement of the corticostriatal synaptic cleft is disrupted in a mouse model of Huntington’s disease

**DOI:** 10.1073/pnas.2210719120

**Published:** 2023-06-06

**Authors:** Carlos Benitez Villanueva, Hans J. T. Stephensen, Rajmund Mokso, Abdellatif Benraiss, Jon Sporring, Steven A. Goldman

**Affiliations:** ^a^Center for Translational Neuromedicine, University of Copenhagen, Faculty of Health and Medical Sciences, Copenhagen N 2200, Denmark; ^b^Department of Computer Science, University of Copenhagen, Faculty of Science, Copenhagen N 2200, Denmark; ^c^Faculty of Engineering, Division of Solid Mechanics, Lund University, Lund 22100, Sweden; ^d^Center for Translational Neuroscience, Department of Neurology, University of Rochester Medical Center, Rochester, NY 14642

**Keywords:** Huntington’s disease, astrocyte, neurodegenerative disease, rabies tracing, synapse

## Abstract

Astroglial dysfunction contributes to the pathogenesis of Huntington’s disease (HD), and glial replacement can ameliorate the disease course. To establish the topographic relationship of diseased astrocytes to medium spiny neuron (MSN) synapses in HD, we used 2-photon imaging to map the relationship of turboRFP-tagged striatal astrocytes and rabies-traced, EGFP-tagged coupled neuronal pairs in R6/2 HD and wild-type (WT) mice. The tagged, prospectively identified corticostriatal synapses were then studied by correlated light electron microscopy followed by serial block-face scanning EM, allowing nanometer-scale assessment of synaptic structure in 3D. By this means, we compared the astrocytic engagement of single striatal synapses in HD and WT brains. R6/2 HD astrocytes exhibited constricted domains, with significantly less coverage of mature dendritic spines than WT astrocytes, despite enhanced engagement of immature, thin spines. These data suggest that disease-dependent changes in the astroglial engagement and sequestration of MSN synapses enable the high synaptic and extrasynaptic levels of glutamate and K^+^ that underlie striatal hyperexcitability in HD. As such, these data suggest that astrocytic structural pathology may causally contribute to the synaptic dysfunction and disease phenotype of those neurodegenerative disorders characterized by network overexcitation.

## Significance

Astrocytic physiological dysfunction contributes to development of the neurodegenerative phenotype in Huntington’s disease (HD), but the structural correlates to this dysfunction are unclear. Here, we used a combination of viral tracing, phenotype-specific tagging, and ultrastructural modalities to reconstruct HD synapses at the nanometer scale in the neostriata of HD mice. We discovered significant impairment in the astroglial engagement of mature striatal synapses. In light of the known deficiencies in glutamate and potassium uptake by HD astrocytes, these findings suggest the potential for leakage of excitatory synaptic contents during neurotransmission, and hence a structural basis for neuronal hyperexcitability in HD. More broadly, our data suggest that astrocytic structural pathology may causally contribute to those neurodegenerative disorders associated with central hyperexcitability.

Huntington’s disease (HD) is a neurodegenerative disorder in which abnormally long CAG repeat expansions in the first exon of the huntingtin (HTT) gene result in a mutant form of the gene (mHTT), which disrupts glial as well as neuronal physiology, leading to dysfunction first evident in neostriatal medium spiny neurons (MSNs) (1). These cells are the only output neurons of the striatum, and receive the vast majority of its cortical and thalamic glutamatergic inputs. As such, the MSNs are the integrating units of the striatum, of which they comprise roughly 95% of all neurons. The glutamatergic inputs of these MSNs receive extensive support from astrocytes, via the synaptic clearance of glutamate and potassium ions by astrocytic processes, as well as by modulatory local gliotransmitter release, which together support the homeostatic maintenance of the synaptic environment (2). As a result, the spatial relationship of astrocytic processes to the individual dendritic spines with which they are associated—and in particular the precise three-dimensional topographical relationship of those processes to the synaptic cleft—may be critical determinants of synaptic efficiency and firing thresholds (3).

The morphology of a dendritic spine is a determinant of its activation dynamics, and hence of its role and importance within that dendritic network (4). The structural relationship of that spine and its associated synapse to the astrocytic processes with which it is engaged is yet another critical determinant of its firing threshold, since the astrocytic processes participate in the sequestration and functional compartmentalization of individual synapses, helping to regulate synaptic activation by removing potassium and glutamate from the synaptic cleft. Yet the structural relationship of synapses with their associated astrocytes has been difficult to study, even moreso to quantitatively describe. Whereas several analytical tools exist for extracting quantitative information from dendritic arbors and spines as captured via fluorescent methods (5–8), analogous tools for structurally rendering nanometer-scale astrocytic interactions with individual dendritic spines have not hitherto been available. Instead, recent strategies have focused on the relationship of synapses to their supporting astrocytic elements, via both fluorescence resonance energy transfer (FRET) microscopy and super-resolution applications in thick tissue (9, 10). In particular, Khakh and colleagues (9) used FRET in an elegant study of the effect of HD on astrocytic interactions with MSNs, concluding that the involution of striatal astrocytes in HD was associated with some astrocytic retraction from affected corticostriatal synapses. Yet this and analogous studies via FRET provide inferential functional data; they do not provide precise or quantifiable structural characterization of the principal elements of the tripartite synapse—the presynaptic terminal, the postsynaptic dendritic spines, and their enveloping astrocytic processes. As such, the relationship of mutant HTT-dependent changes in dendritic spine morphology, to changes in astrocytic structure, and the effects of these changes on synaptic structure and function, has remained unclear. More broadly, such topographic assessment and nanometer-scale reconstruction of perisynaptic environments have remained a challenge, especially so in the adult mammalian brain.

To address this issue, in this study we combined correlated light electron microscopy (CLEM) with serial block-face scanning EM, to directly visualize specific astrocyte–synapse interactions on striatal MSN spines, and then developed an analytical strategy by which to extract quantitatively significant information from nanometer-scale structures in these discrete 3D volumes. We combined monosynaptic retrograde tracing using glycoprotein-deleted replication-incompetent rabies virus (11), with astrocyte-specific lentiviral tags to visualize those astrocytes interacting with the targeted synapses. This approach allowed us to define the structural components of synaptic fields in these brains at resolutions of <10 nm, over volumes of 9,000 µm^3^. We chose to study the neuronal phenotype revealed by retrograde tracing of MSNs via their projections to the globus pallidus external segment (GPe), since GPe projection neurons are predominantly D2 receptor-expressing MSNs, and are the first striatal neurons to manifest dysfunction in HD (12). We found that in HD mice, the synaptic environments of mature dendritic spines experience a marked decrease in both astrocytic engagement and synaptic isolation, suggesting a disease-associated decline in the contribution of astrocytes to both synaptic function and sequestration in the adult brain.

## Results

### Astrocytic Engagement of Single Corticostriatal Synapses May Be Directly Visualized.

**Fig. 1.**
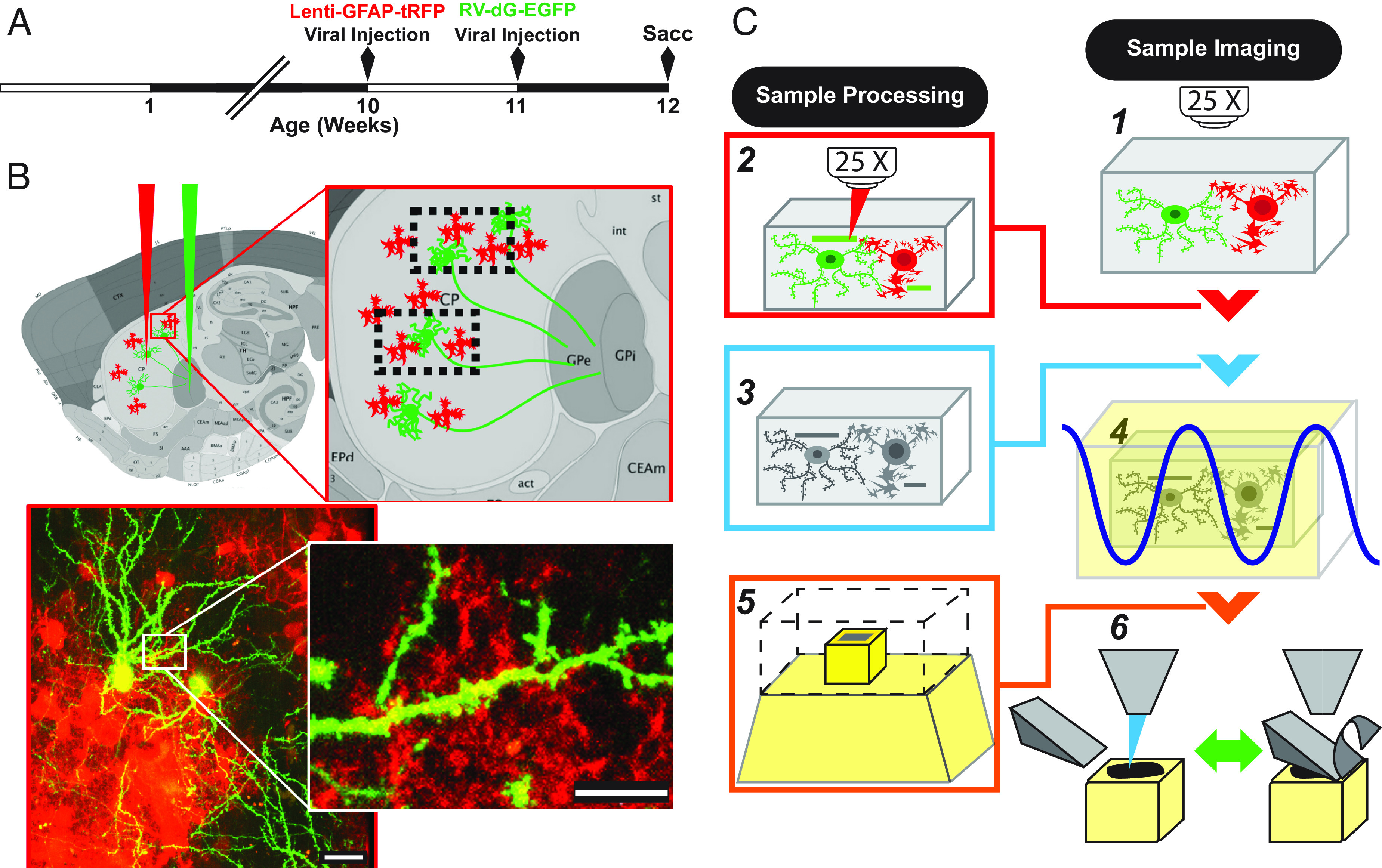
Phenotype-specific ultrastructural analysis of synaptic–glial interactions using SBF SEM. Rabies tagging of neuronal pairs was combined with lentiviral GFAP-GFP-based identification of coupled astrocytes, as assessed by correlated light EM (CLEM) and serial block-face scanning electron microscopy (SBF SEM), to describe prospectively defined astrocyte-engaged synaptic fields. (*A*) Timeline of sequential viral deliveries. (*B*) Astrocyte-engaged synaptic fields identified by pallidal rabies retrograde tagging of the MSN neuronal network combined with astrocyte-specific Lenti-GFAP-tRFP. (*C*) Workflow by which sequential multimodal imaging is used to pinpoint ROI: 1) 2-photon imaging is used to identify interacting domains; 2) 2-photon branding is used to induce near-infrared ablations in the vicinity of the ROI; 3) EM sample preparation via dehydration, osmium impregnation, and resin embedding; 4) microCT scan of the entire sample; 5) relocalization of ROI via microCT cross-reference and trimming of excess material; and 6) serial block-face SEM of the ROI domain. (Scale: 10 µm)

We used CLEM (13) together with 2-channel fluorescence imaging of turboRFP (tRFP)-tagged, retrograde traced MSNs and lentiviral EGFP-tagged astrocytes to reveal interacting domains of striatal MSN dendrites and astrocytic processes in the adult mouse neostriatum (Fig. 1). This combination of imaging modalities allows the capture of relevant light microscopy data prior to serial EM scans, facilitating retrieval of the desired regions of interest (ROI) in EM and preserving contextual information of the nanometer-scale, serial SEM datasets (Fig. 1*C* and *SI Appendix*, Fig. S1). Using this approach, we first captured EM datasets of specific MSN dendritic spines displaying astrocytic processes in close proximity to the periphery of the postsynaptic density (PSD). We followed this with a quantification strategy by which we assessed the global spatial engagement of astrocytic processes cradling the synaptic environment, and the effects of HD on these structures.

To that end, we injected GFAP-tRFP lentivirus bilaterally into the striata of R6/2 × Rag1^−/−^ or WT Rag1^−/−^ control mice at 10 wk of age, followed a week later by intrapallidal injection of replication-inefficient rabies virus with EGFP substituted g-protein (RV-dG-EGFP) (Fig. 1*A*). (Of note, we used immunodeficient Rag1^−/−^ and R6/2 × Rag1^−/−^ mice because of our long-term interest in assessing the effects of human glial progenitor cell transplantation as a means of astrocytic replacement in this model, and hence as a therapeutic strategy. For these still-planned experiments, immunodeficient hosts will be required for graft acceptance. To enable the future comparison of astrocytic engagement in hGPC-transplanted R6/2 mice with the untransplanted mice of the present study, we therefore used Rag1^−/−^ mice here as well.) A week after rabies injection, at 12 wk of age, we killed the mice and used correlated light EM (CLEM) to isolate synaptic regions of interest (ROIs), focusing on regions with predefined astrocytic processes intermingling with dendritic arbors of MSNs. These regions were identified, ablated, and selectively isolated through correlative methods (Fig. 1*B*), culminating in serial SEM acquisition for nanometer-scale data acquisition (Fig. 1*C*, *SI Appendix*, Fig. S1, and Movie S1). Data were collected from striatal sections sampled from 2 WT and 3 R6/2 animals. We focused on dendritic branches of at least 3rd order, and only dendritic spines that displayed astrocytic engagement; only those spines engaged by astrocytes were included in this analysis. Of the 144 total astrocyte–synapse ROIs we selected, 22 did not present astrocytic apposition within the first 100 nm of the radius surrounding the PSD once the SEM series were segmented, and thus were not included in this analysis. The remaining 81% of the ROIs (117 astrocyte–synapse ROIs) were analyzed for various metrics, indicating a modest success rate for capturing the astrocytic interactions of those synapses exhibiting close neuron–glia engagement.

We observed a variety of structural configurations with regard to relationships of astrocytic processes engaging synapses, both presynaptically and postsynaptically. To standardize our quantitative descriptions of these synapses, we focused on their postsynaptic densities. We identified the postsynaptic densities as regions on the head of the dendritic spine presenting high-electron-dense material in the EM datasets, as expected for asymmetric glutamatergic synapses. Once we identified prospective ROIs, defined as regions within which astrocytic domains revealed by tRFP engaged the EGFP-expressing dendritic spines of striatal MSNs, we imaged those volumes containing the selected ROIs. We then selectively ablated points within that ROI with high-power IR laser scans, so as to generate autofluorescent, spatially discrete fiducial features (Movie S2) (14). From these ROIs, we traced specific segments across 3 orthogonal imaging scales (2-photon, X-ray tomography, and serial EM), and then correlated the corresponding datasets so as to acquire the tripartite synapses found within the original ROI (Movie S2). By this means, we were able to directly visualize our prospectively defined synapses of interest.

### Astrocytic Proximity and Peripheral Coverage of Synapses Is Disrupted in R6/2.

**Fig. 2.**
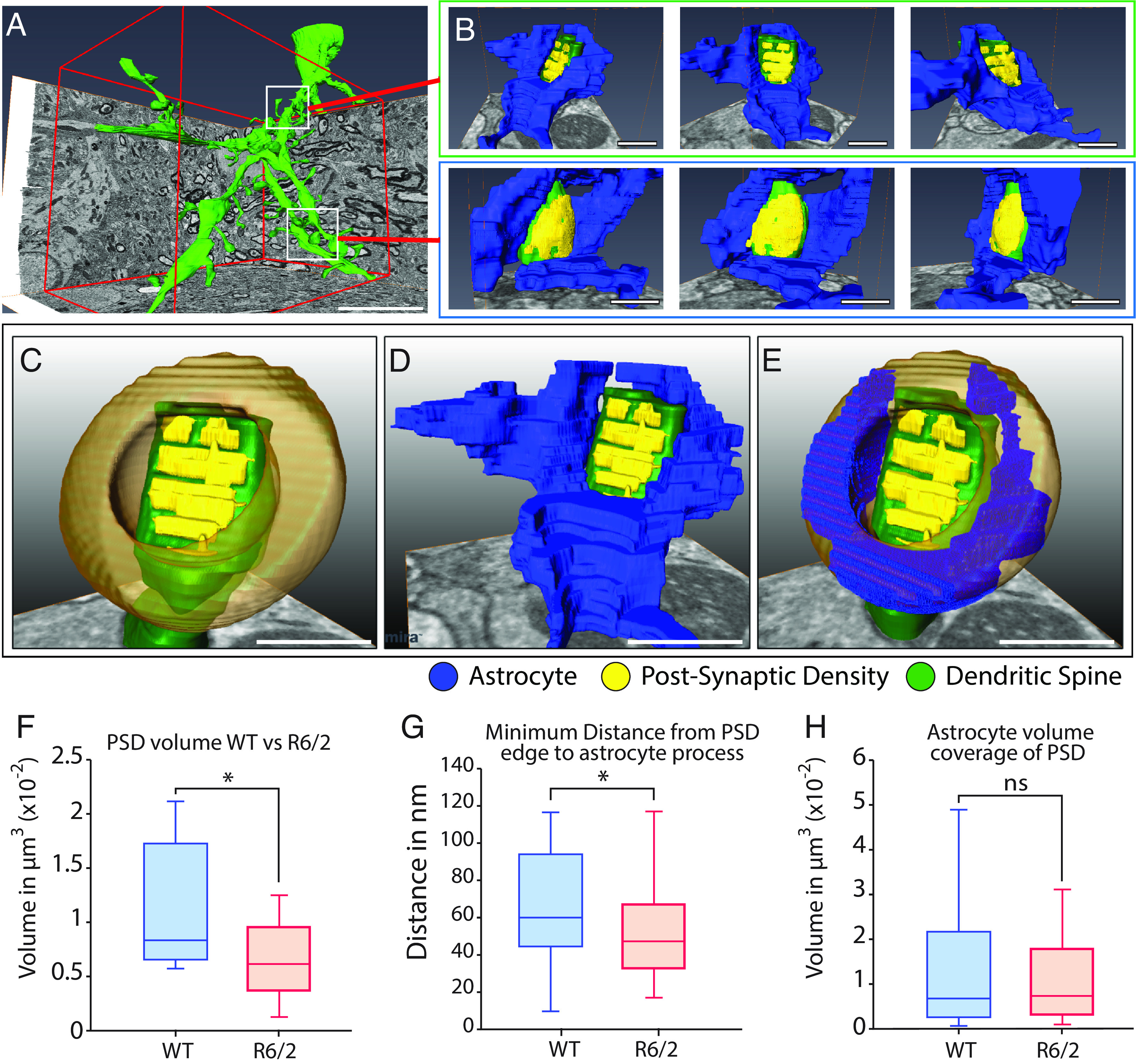
Analysis of astrocyte–synapse interactions. (*A*) Detailed view of dendritic branch and spine reconstruction. (*B*) Reconstruction of selected spines and perisynaptic astrocytic domains in 3D, with three views around the axis for each spine (spines, *green*; postsynaptic densities, *yellow*; peripheral astrocyte domains, *blue*). (*C*) Definition of analytic boundaries of the torus region parallel to the PSD, with outer bounds defined as R_outer_bound = R_max_ (of PSD) * 1.5 and R_inner_bound = R_max_ * 0.75. Depth/thickness of the torus, 300 nm. (*D*) Astrocyte domain contained within the periphery of the PSD (blue, transparent). (*E*) Exclusion of excess material contained outside the bounds of the analytical torus and highlight of the astrocyte domain (*blue*, opaque) within the total analysis domain (*orange*, transparent). (*F*–*H*) All data were limited to the predefined analytic domains. (*F*) Volume of PSD (nonequal variances, *t* test two tailed, *P = 0.03). (*G*) Minimum distance from PSD edge to astrocyte process edge, nonequal variances (*F* test 0.009, *t* test *P = 0.04). (*H*) Astrocyte coverage within a radius 1.5 * r_max_ of PSD. Equal variances, nonsignificant (*P* = 0.324, n.s.) (WT: two mice, n = 32; R6/2: 3 mice, n = 45). (Scale: 5 µm)

We next postulated that for regions of interacting astrocyte processes and MSN dendritic domains, the degree of infiltration of the synaptic periphery by the astrocyte would be affected by HD-associated pathology. To test this postulate, we relocalized our ROI, identified the corresponding dendritic domain, and then manually segmented those spines engaged by astrocytic processes (Fig. 2 *A* and *B*). We generated regions of analysis that incorporated features derived from the PSD, modeling that region on a torus structure surrounding the synaptic cleft (Fig. 2*C*). We set the bounds of analysis as a torus-shaped region surrounding each PSD so as to normalize the 3-dimensional variability of these structures within and across datasets. Specifically, we measured the longest axis of the PSD structure and defined the inner and outer bounds of the virtual torus surrounding the PSD as 0.75 * R_max_ and 1.5 * R_max_, respectively, so as to establish a consistent link between the region analyzed and PSD morphology. The depth of the torus was set to 300 nm, as the thickness of each PSD was consistent, regardless of the PSD area on the dendritic spine head; the center of the virtual torus was set to the 3D barycenter of the PSD. Anything outside of this torus was excluded, and we captured astrocytic data only within the torus-like structure for quantification. By this approach, we defined the peripheral limits of analysis, while normalizing the variability across PSD configurations (Fig. 2 *D* and *E*); we quantified the volume of the PSD itself, the minimum distance between the PSD edge and the astrocyte membrane, and the total volume of astrocytic cytoplasm for each synapse within each segmented synaptic environment (Fig. 2 *F–H*).

We found that the volume of the segmented PSD was significantly lower in R6/2 mice than WT mice, with an average of 10.9 ± 1.6 × 10^−3^ µm^3^ for WT and 6.3 ± 0.9 × 10^−3^ µm^3^ for R6/2, spanning a range of 5.6 to 21.1 × 10^−3^ µm^3^ for WT and 1.2 to 12.8 × 10^−3^ µm^3^ for R6/2 (Fig. 2*F*; *P* < 0.05, two-tailed *t* test; WT, n = 32 synapses, R6/2, n = 43). We also found the minimum distance from the PSD boundary to the astrocytic process edge to be significantly reduced in R6/2 synapses—indicating closer proximity—with an average distance of 73.5 ± 8.1 nm for WT vs. 58.7 ± 4.5 nm for R6/2 (*P* < 0.05, WT: n = 32 synapses; R6/2: n = 45 synapses; Fig. 2*G*). However, despite an apparent reduction in the astrocytic coverage of the synaptic periphery in R6/2 mice, this initial approach did not reveal an overall reduction in perisynaptic astrocytic volume in R6/2 mice, relative to their WT controls (Fig. 2*H*, *P* > 0.05). Rather, this analysis indicated that HD R6/2 mice exhibit diminished PSD size, with closer apposition of astrocytic processes to the PSD. In that, these data support the aforementioned FRET study of Khakh and colleagues (9), which also observed closer apposition of astrocytic processes to striatal synapses in HD mice than in WT controls. To resolve this apparent paradox of closer astrocytic proximity yet diminished areal coverage of R6/2 synapses, we next sought to parse out the detailed topographies of HD and WT MSN synapses, focusing on both the detailed astrocytic geometry at their respective synaptic clefts and on the types of synapses—and hence the structural maturity of the dendritic spines—with which those astrocytes are engaged.

### Astrocytic Infiltration of the Synapse Is Altered as a Function of Both HD and Spine Class.

**Fig. 3.**
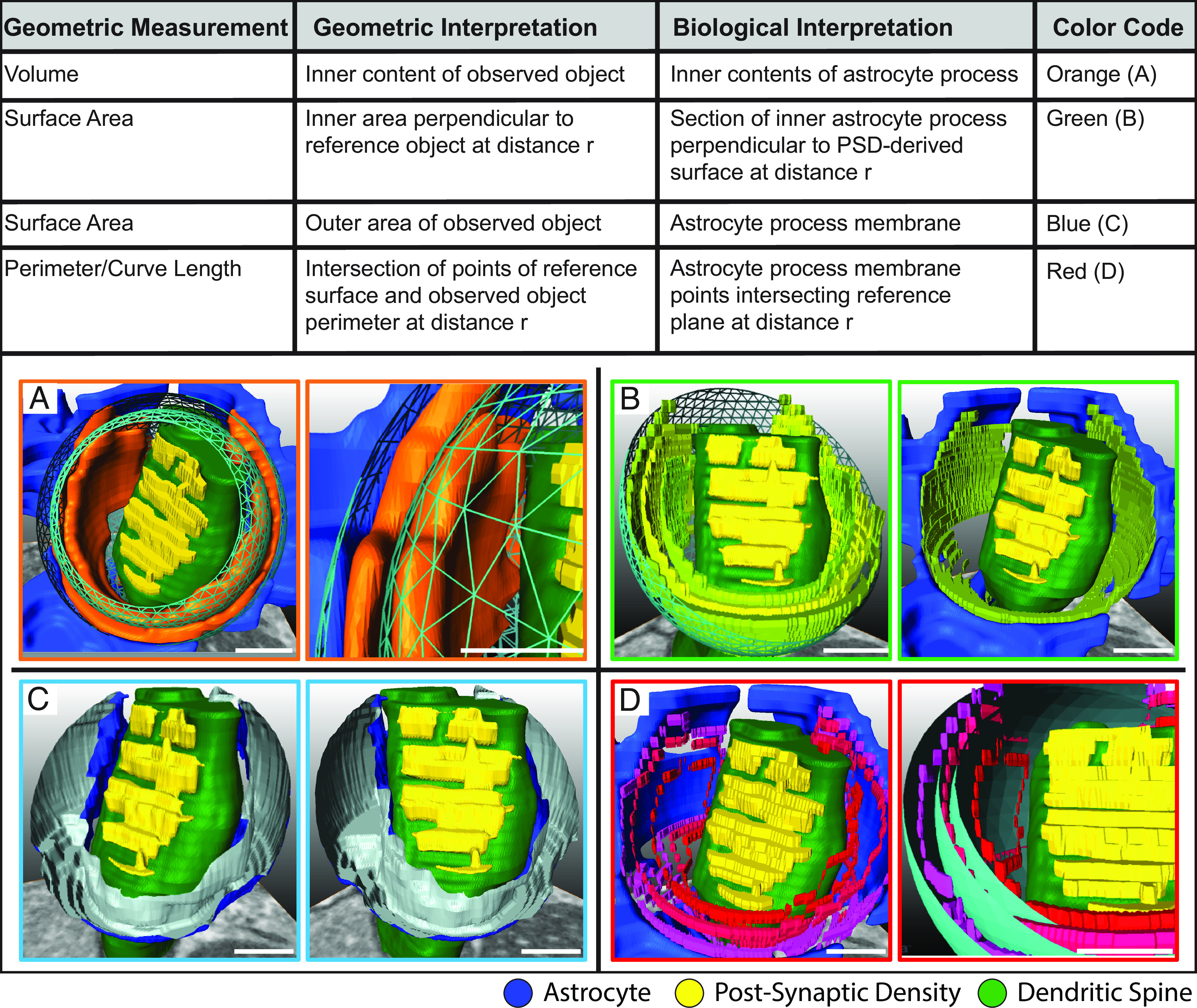
Modeling the topographic relationship of astrocytes to dendrites at the synapse. To model the topology of astrocytic synaptic engagement, we used a spherical approximation of geometric measurements, defined as at the intersections of interacting reference and observed surfaces, at increasing distance (radius) from the PSD. To this end, we assessed the 3D interactions of the astrocytic processes with the synapse and derived geometric measurements (Hausdorff measures) for each astrocyte process (observed object). These geometric derivations included astrocyte surface area (in *blue*), volume (*orange*), surface area perpendicular to the reference object (*green*), and intersection points (contour) in *red*. (*A*) Representative view of astrocyte volume surrounding the PSD (*orange*, astrocytic cytoplasm) measured over discrete measurements represented by teal intervals surrounding the PSD. (*A*, *Left*), overview; (*A*, *Right*), identification of discrete intervals and quantified material. (*B*) Discrete measurements of the astrocyte surface area perpendicular to the PSD (*Left*) shown at increasing radii intervals from PSD. Excluded astrocyte material shown in blue (*Right*) (*C*) Surface area of the astrocyte surrounding PSD within the predefined analysis limits (*blue*, corresponding to the cell membrane). View from two angles. (*D*) Contours where the astrocyte structure intersects the PSD-derived surface. Multiple iterations of contours at intervals (*D*, *Left*). Contour overlay shows measurement steps in *teal* (*D*, *Right*). (Scale: 250 nm)

To thus describe the structural relationships between individual synapses and their partnered astrocytes, we established a model by which the topographies of individual synapses and their astrocytes could be accurately rendered and quantitatively described. For each modeled synapse, we measured the astrocytes in a progressively larger region around the PSD. These extended regions can be understood as a dilation or inflations of the PSD, or more formally, as the set of all points within a progressively increasing distance from the PSD edge. This forms a set of measurement functions defining the geometrical features of the astrocyte as a function of the distance to the PSD. As such, each synapse contributes a set of measurement functions which, taken together, can be understood as a cross-K-function summary statistic (15). By this means, we analyzed the interactions of the astrocytic processes as they intersected the virtual expansions of the PSD. In particular, this method allowed us to estimate the astrocyte volume and surface area as a function of the distance to the PSD (Fig. 3) (16). By this approach, as the PSD is dilated, the points and characteristics of the dilation’s interaction with the surrounding astrocyte are recorded.

Past studies of astrocytic calcium dynamics have reported the derivative of the astrocytic volume to be highest within 200 nm of the postsynaptic densities (17). In addition, simulations of extrasynaptic glutamate receptor (GluN) diffusion by Gavrilov and colleagues have suggested that this distance covers the glutamatergic activity profiles important to LTP and LDP dynamics (18). On that basis, we chose to focus on, and analyzed, the geometric characteristics of the astrocyte processes within a 150-nm range spanning 25 to 175 nm of the PSD.

A number of studies have reported that the astrocytic coverage of central synapses is linked to spine maturity (19–22). We therefore categorized our observations on astrocytic interactions with synapses on the basis of dendritic spine morphology and inferred maturity (Movie S3). In particular, we assessed the astrocytic engagement of separately identified subpopulations of thin and mushroom spines, which roughly correspond to immature and more mature dendritic spines (23–26). We separately validated these different categories of spines utilizing k cross-section statistical methodology (*SI Appendix*, Figs. S2 and S3) (16). Using this analytic strategy, we then asked whether the apposed astrocytic volume associated with each synapse was significantly altered in HD mice, and whether this varied as a function of spine maturity.

**Fig. 4.**
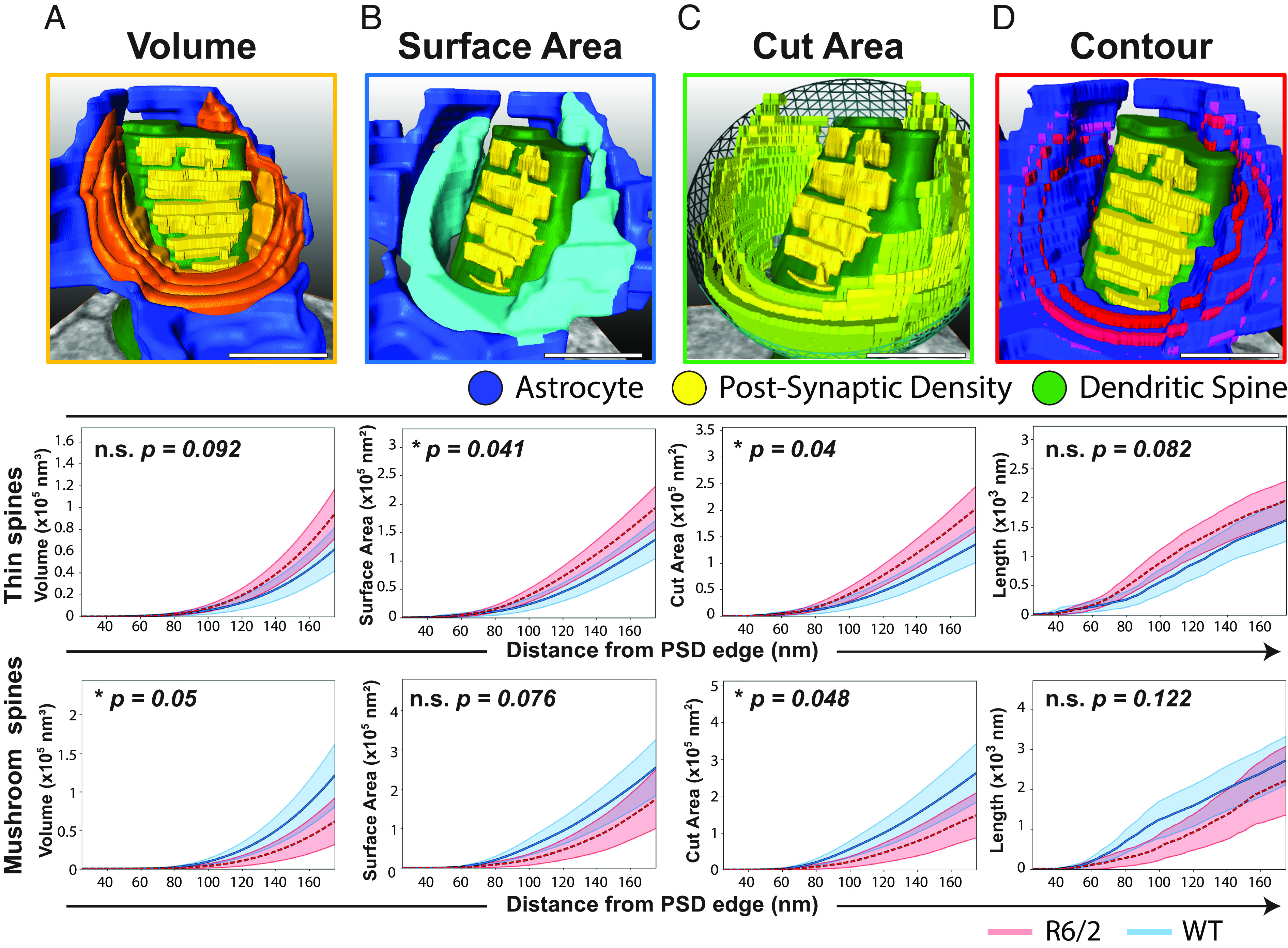
HD affects the geometry of astrocyte–synapse interactions. Analysis of astrocyte–synapse interactions using a derivation of k cross-section statistics. Perisynaptic astrocytic volume, surface area, perpendicular area, and contour length were each measured over the proximal interval of 25 to 175 nm from the PSD edge; cumulative data shown. (*A*) Perisynaptic astrocytic volume Thin/immature spines, with a trend to increased associated astrocyte volume in R6/2 relative to WT (*Top* panel, *P* = 0.09, n.s.). Mature/mushroom spines, with significantly decreased associated astrocytic volume (*Bottom*, *P = 0.05). (*B*) Surface area Thin immature spines of R6/2 mice were engaged by an increased surface area of their associated astrocytes (*Top* panel, *P = 0.04). In contrast, the neighboring astrocytes of mature/mushroom spines, trended to present a decreased surface area to the synapse in R6/2 mice (*Bottom*, *P* = 0.08, n.s.). (*C*) Cut area Thin/immature spines, increased neighboring astrocyte perpendicular surface area (*Top* panel, *P = 0.04). Mature/mushroom spines, decreased neighboring astrocyte orthogonal surface area (*Bottom*, *P = 0.048). (*D*) Contour Thin/immature spines, unchanged neighboring astrocyte contour length (*Middle* panel, *P* = 0.082, n.s.). Mature/mushroom spines, unchanged neighboring astrocyte contour length: (*Bottom* panel, *P* = 0.12, n.s.). Thin/Immature spines: WT, four mice, n = 30 spines; R6/2, 5 mice, n = 35 spines. Mature/mushroom spines: WT, four mice, n = 27 spines; R6/2, 5 mice, n = 22 spines. Monte Carlo permutation method, absolute AUC, 95% CI shown. (Scale: 500 nm)

To this end, we first measured the astrocyte volume in the periphery of the synapse and found significant atrophy on mature, mushroom-like synapses (Fig. 4*A*). In particular, mushroom spine synapses displayed a significant decrease of engaged astrocytic volume, both in cumulative terms and upon distance-normalized analysis (Fig. 4*A* and *SI Appendix*, Fig. S4 *A*, *Bottom*). In contrast, we found a trend toward increased astrocytic volume on thin spine synapses, both in their cumulative or distance-normalized volumetrics (Fig. 4*A* and *SI Appendix*, Fig. S4 *A*, *Middle*). Again analyzing within 175 nm of the PSD edge, we found that the average volume of astrocyte coverage of mushroom spines in R6/2 was 7.4 ± 0.17 × 10^−4^ µm^3^ vs. 13.7 ± 0.2 10^−4^ µm^3^ for WT. In contrast, R6/2 thin spines displayed astrocyte volumetric engagement of 10.6 ± 3.3 × 10^-4^ µm^3^ vs. 6.9 ± 1.3 10^−^^4^ µm^3^ for WT controls. Together, these data indicate that mutant HTT expression in R6/2 mice is associated with a substantial diminution of astrocytic apposition to synapses on mature spines of striatal MSNs, but no such loss of astrocytic engagement with immature, thin spines within the same striata.

### The Perisynaptic Astrocytic Surface Area Is Altered in HD Differentially by Dendritic Spine Type.

We next asked whether R6/2 astrocyte processes exhibited disease-dependent changes in their perisynaptic surface area and whether this too might vary as a function of spine class. To this end, we analyzed our structural data as a function of both the distance from the PSD boundary and of spine class (Movie S4). We found that mushroom spine synapses exhibited a strong but less than significant trend toward a diminished astrocytic surface area cradling the synaptic cleft, as might be predicted on the basis of the sharply decreased astrocytic volume associated with these synapses (*P* = 0.076) (Fig. 4*B* and *SI Appendix*, Fig. S4 *B*, *Bottom*). In contrast, we found that the surface area of astrocytic processes engaging thin spines was increased in R6/2 MSNs, relative to controls (*P* = 0.041) (Fig. 4*B* and *SI Appendix*, Fig. S4 *B*, *Middle*). In particular, the average surface area of astrocyte coverage upon mushroom spines (within 175 nm of the PSD edge) in R6/2 was 1.93 ± 0.47 × 10^−5^ µm^2^ vs. 2.75 ± 0.53 × 10^−5^ µm^2^ for WT. In contrast, R6/2 thin spines encountered perisynaptic astrocyte surface areas of 2.08 ± 0.66 × 10^−5^ µm^2^ vs. 1.5 ± 0.27 × 10^−5^ µm^2^ for WT mice. Overall, these results indicate differential effects of mHTT expression on astrocytic membrane geometry and engagement on thin and mushroom spines, with the loss in R6/2 mice of astrocytic membrane availability within the vicinity of mature, mushroom spine synapses, despite the concurrent increased astroglial coverage of thin spines, as assessed by surface area.

Among the derivations from the cross-K statistic methodology, we also quantified the cut area and contour extension of the intersecting objects (i.e., dilated PSD reference object and astrocytic process). While surface area and volume are metrics with clear biological identities, perpendicular area and contour are derived functions. Nevertheless, we found the astrocyte perpendicularly cut area to be significantly decreased for mushroom spines (Fig. 4 *C*, *Bottom*) and increased for thin spines (Fig. 4 *C*, *Middle*). Conversely, the contour measurements displayed no significant difference when we compared the astrocytic coverage of thin vs. mushroom spines (Fig. 4*D*).

Over the perisynaptic distance interval studied, we were presented with the difficulty that cumulative comparisons and differences can be disproportionately inflated as a function of increasing radius. Much as the area of a circle increases proportional to the square of the radius, the surface area of a spherical domain of interest increases by the cube of the radius from the PSD. This exponential increase in surface area can be controlled for by normalizing the data, which considers the recorded cumulative information in comparison with the exponential increase per radius step. This normalization is analogous to converting the total astrocyte coverage to proportional coverage. When these data were normalized to radial distance from the PSD, the disease (R6/2 vs. WT)-dependent atrophy of astrocytic processes associated with mushroom spines was sustained across all geometric metrics (*SI Appendix*, Fig. S4).

Of note, our classification of thin and mushroom spines was verified using the same method of k cross-section statistics, setting the observed object as the spine rather than its associated astrocyte. By this means, we found that while the surface area and volume of the two spine classes—thin and mushroom—differed from one another, they did not differ significantly between WT and R6/2 mice (*SI Appendix*, Fig. S3).

## Discussion

In this study, we developed a methodology for quantitatively assessing the structural relationships of astrocytes to their partnered synapses, and for doing so as a function of glial disease. In particular, we designed two complementary approaches to generate spatial information sufficient to describe the geometry of synaptic environments. The first involved the modeling of a torus region, defined by the size of the PSD, over which several relationships between the PSD and the astrocyte could be quantified. The second strategy used k cross-section statistics to derive the 3D geometric qualities of the astrocytic engagement into discrete, quantifiable parameters. This image-based analytic protocol provided us a means of assessing and quantifying the structural correlates to both normal and pathological synaptic function. In this study, we employed this methodology to assess the effects of HD on the astrocytic engagement of striatal synapses.

Using this strategy, we found that astrocytic engagement of corticostriatal excitatory synapses on MSNs is significantly impaired in the R6/2 mouse model of HD. This disease-associated defect in glial synaptic engagement was manifested by abnormalities in astrocytic sequestration of the PSD, as reflected by astrocytic retraction from mature dendritic spines, despite paradoxically increased astrocytic apposition to thin, immature spines. These data indicate that while the astrocytic engagement of glutamatergic synapses varies with spine maturity, the structural relationship of astrocytes to their partnered synapses in the HD striatum is abnormal at multiple stages of spine maturation.

While our data were consistent with regard to the aberrant relationships of astrocytes with their partnered synapses in HD mice, and of the prominence of this effect throughout postsynaptic spine maturation, our use of T- and B-cell-deficient Rag1^−/−^ hosts—done so as to permit later comparisons of synaptic structure in human glial-transplanted HD hosts—poses a potential caveat to this study. While T cells are typically sparse in the normal adult brain parenchyma (27, 28), entering primarily in the setting of active inflammation or injury (29), increased T-cell surveillance in the cerebrospinal fluid of HD patients has been reported (30). This is notable as T-cell function has been implicated in the regulation of synaptic structure, possibly via interactions with microglia during developmental synaptic pruning (31–35), suggesting the possibility that the T-cell deficiency of Rag1 null mice might indirectly influence aspects of synaptic structure. That said, in the present study, both the wild-type (WT) and R6/2 mice shared the same Rag1^−/−^ immunodeficient background, thereby isolating the specific effect of mHTT expression to the astrocytic-synaptic interaction.

**Fig. 5.**
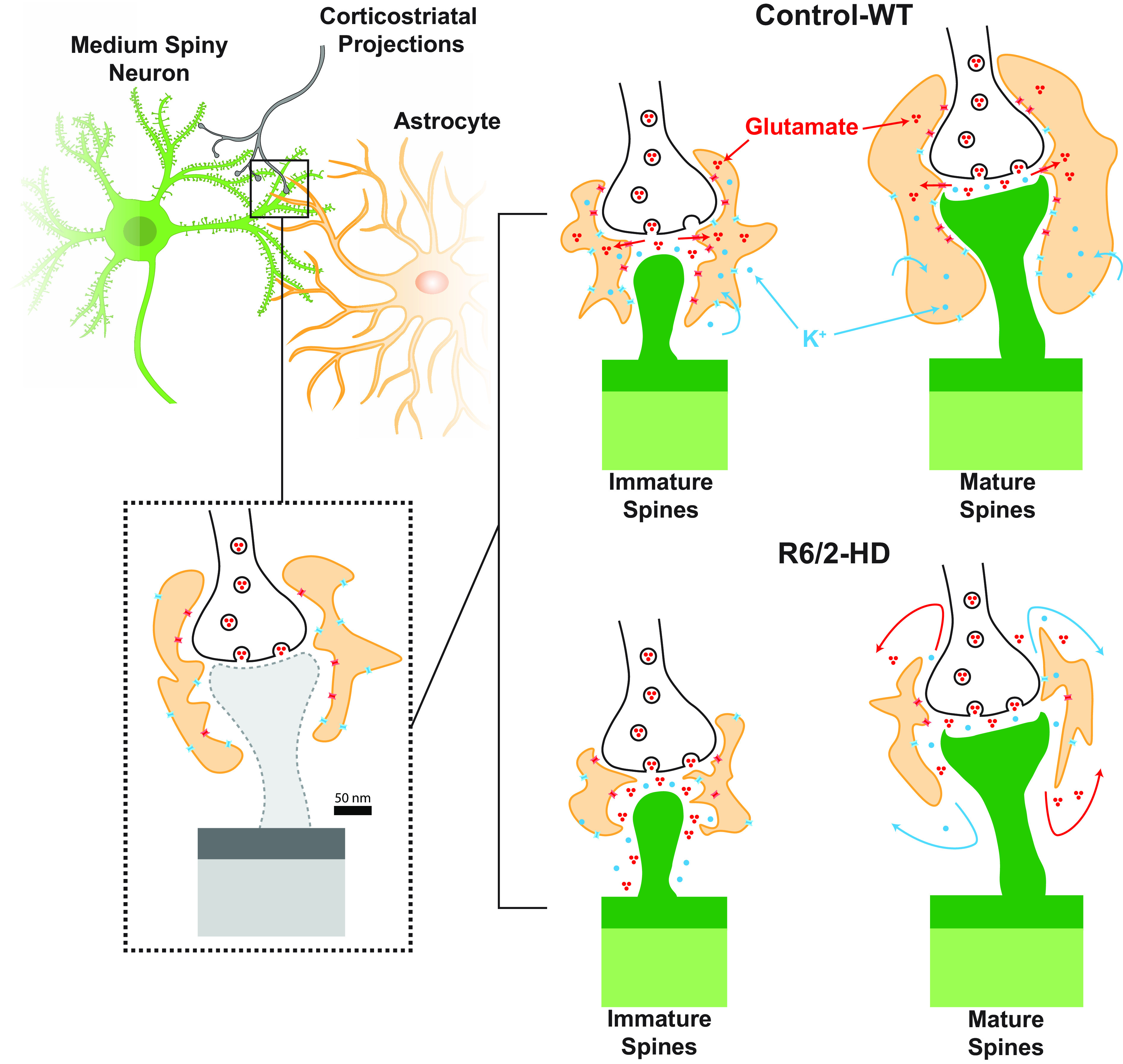
Differential disease-related changes in the astrocytic coverage of mature and immature dendritic spines. Thin immature spines of young adult mice display increased engagement of synaptic periphery in R6/2 mice, increasing sequestration of glutamate and ions. In contrast, mature spines experience significant retraction of astrocytic processes from the synaptic periphery, exacerbating the leakage of K^+^ and Ca2^+^ ions as well as glutamate, which together contribute to nonspecific signaling and activation of extrasynaptic receptors.

Several recent studies of HD mice have noted a disease-dependent reduction in astrocytic domain size and in the number of close proximity synapse–astrocyte sites (9, 36). In the present study, we sought to more precisely define the effects of HD on synaptic ultrastructure, by combining rabies viral tagging of defined synaptic pairs and lentiviral EGFP tagging of their partnered astrocytes, with electron microscopy to establish a correlative light-electron microscopy (CLEM)-based approach to study the astrocyte-synapse interactions between specific, prospectively identified presynaptic and postsynaptic neuronal partners. In particular, we combined rabies tagging of corticospinal-MSN synapses with lentiviral GFAP-driven EGFP identification of local astrocytes and examined the resultant tissue by sequential imaging using 2-photon, X-ray, CLEM, and serial block-face scanning EM. By this means, we were able to investigate the structural topography of corticospinal-MSN synapses together with their partnered astrocytes with a never-before-achieved level of resolution and specificity. In thereby resolving the synaptic environment, we were surprised to observe that these different spine morphologies were associated with such different degrees of astrocytic coverage. This was unexpected, as recent studies had suggested that spine size per se does not determine the astrocytic volume fraction in the synaptic microenvironment (18). Nonetheless, we found that HD astrocytes exhibited increased apposition to thin, nominally immature spines, while more mature, mushroom-like spines enjoyed significantly less astrocytic engagement. As such, these data indicated that the relationship of astrocytes to synapses of different spine maturational classes varies with the disease environment (Fig. 5).

In this regard, we found that while mHTT-expressing astrocytes maintained their engagement with immature spines, the effects of astrocytic involution were more profound on those resident neurons with more mature spines, which suffered both astrocytic retraction from and desequestration of their embedded synapses. Indeed, the maturity-dependent decline in astrocytic engagement of striatal synaptic spines, despite retained glial fiber proximity, suggests that R6/2 astrocytes become atrophic and that this process becomes most manifest in the most mature synapses, identified by the mushroom-like morphologies of their postsynaptic spines. As such, their distal processes might maintain proximity, but more broadly, they lose volume within the perisynaptic region; as a result, the mature spine synapse loses its astrocytic investment and hence its spatial sequestration. In contrast, the close relationship of astrocytes to synapses along thin R6/2 spines appears to reflect a sustained, if not actually tighter, engagement of the synaptic periphery by R6/2 astrocytic processes. Yet, since HD glia are deficient in K^+^ and glutamate uptake (37), these thin spine synapses might be expected to suffer increased intrasynaptic K^+^ and glutamate levels even with sustained spatial sequestration (Fig. 5), hence contributing further to the hyperexcitability of striatal neuronal networks in HD. Overall, the abnormal patterns of astrocytic engagement with both mature and immature spines, and their attendant alterations in the perisynaptic sequestration of both neurotransmitters and neuroactive ions, would be expected to degrade input-specific responses, confounding LTP and LDP dynamics at the cellular level (17, 18), while disrupting network coordination at the systems level.

Although the highly branched, bushy structure of astroglial processes makes the astrocyte a difficult domain to study structurally, its functional role in the maintenance of synaptic homeostasis can be leveraged to infer fine morphological changes. In that regard, a recent study by Khakh and colleagues exploited the phenomenon of Förster fluorescence resonance energy transfer (FRET) to study the points of closest proximity of astrocytic processes to synapses (9). To do so, they selected and modified presynaptic and perisynaptic components to express mCherry and GFP, respectively. They then induced fluorescence by one fluorophore and recorded emission events from the other, capitalizing upon the occurrence of two-color emissions only in those regions where the fluorophores are in close enough proximity for FRET events. Yet, while elegant, FRET events, at 50% efficiency, only occur at those sites where GFP and mCherry localize within 5.1 nm of each other; in general, FRET is sensitive to changes over 10 nm, where its efficiency ranges from 5% to 95%. Thus, while this method reveals the existence of close proximity interactions between astrocytic and neuronal structures, it is a semi-binary readout, providing information primarily on the existence—or absence—of synapse–astrocyte interactions within 10 nm of each other. FRET does not provide information on the characteristics of these interactions on an individual spine-by-spine approach, nor does it provide information regarding the astrocyte interaction beyond 10 nm. In similar studies by Gavrilov et al., analyzing astrocytic coverage of synapses in the hippocampus, the investigators expanded the analysis volume to 600 nm from the PSD. According to their postsynaptic receptor activation simulations, the open probability of extrasynaptic GluA receptors peaked at r = 0.15 µm (from PSD edge), while GluN receptors seemed to display a generally higher peak than GluA, but decayed more steeply (18). These data suggested that the region of astrocytic engagement most relevant to synaptic function lies within ~200 nm of the PSD; as such, we chose to investigate the geometries of perisynaptic astrocytic processes within that range.

Recent studies have highlighted the contribution of glial pathology to HD (9, 36–43). These studies have pointed out that mHTT-associated astrocytic dysfunction in particular may have widespread consequences on neuronal as well as glial function. These effects include a disruption in synaptic K^+^ uptake (36), with insufficient clearance at synapses (44); neuronal hyperexcitability due to high intrasynaptic glutamate as well as K^+^, with attendant excitotoxicity (45); and an overall reduction of the astrocytic domain size and hence in synaptic coverage (9, 36, 38, 44, 46). Parallel studies have highlighted the relationship of astrocytic engagement to spine size and maturity (18, 22, 47, 48); this issue is of particular relevance here due to the scaling of AMPA receptor content to PSD size, and therefore to spine maturity (49–53). In the present study, we extended these observations by comparing the astrocytic engagement of striatal synapses in HD transgenic and wild-type brains, focusing on the effect of HD on the relationships of perisynaptic astrocytes and their processes to single striatal synapses.

As in our structure-based study of synaptic topologies in HD, Khakh and colleagues’ recent FRET-based study discussed above noted an overall reduction in the R6/2 HD striata in the volumes of astrocytic domains, despite the closer proximity of those glial–synaptic interactions that did occur (9). Yet such measures tell an incomplete story: R6/2 striatal MSNs experience a significant reduction in both their dendritic arbors and in the density of their dendritic spines (12). Furthermore, the proportion of immature to mature spines on the remaining dendrites of 12-wk-old R6/2 MSNs is markedly increased, suggesting that most of the functional spines remaining in these mice are thin and relatively immature (54). Together, these data suggest that FRET readouts from the R6/2 HD striatum may derive predominantly from thin spines, which—unlike mature spines—may manifest closer astrocytic engagement, that appears transient and may be compensatory, but which is any case lost with spine maturation. As such, the reduced glial engagement of mature R6/2 synapses, while still clear and significanti, may nonetheless be artifactually minimized in our overall dataset owing to the relative under-representation of mature synapses in the late-stage R6/2 striatum (54, 55).

In this regard, the stage-dependent variability in the shapes of presynaptic and postsynaptic elements introduced a challenge from the perspective of quantitative standardization, as we needed to define a reference frame consistent across all synapses surrounded by astrocytic processes. While the structures of axon terminals and dendritic spines have predictable shapes, their sizes and engagement geographies vary such that the center of the spine head may occur at comparatively different distances from the spine–axon interface. We therefore chose to center our quantitative analysis on the postsynaptic densities, as comparatively flat, disc-like structures that are invariably found in close proximity to a presynaptic terminal, thus ensuring that our measurements of astrocytic structure specifically describe engagement at the synaptic periphery. Doing so established the reduced, more distant astrocytic circumvention of mature R6/2 synapses, while allowing our analysis to be quantitatively independent of the exact position of the vesicle release points of presynaptic terminals.

There is mounting evidence that morphological changes in neuroglial interactions are both malleable and activity dependent. For example, the induction of long-term potentiation (LTP) in hippocampal spine terminals leads to the mobilization of neighboring astrocyte processes within 5 min of LTP induction (56). Other recent studies have highlighted the complex nature of the signals between neurons and glia that cooperatively regulate synapse formation and maturation (3). In that regard, while our work did not explore the molecular mechanisms by which astrocytic engagement of synapses is affected in R6/2 mice and in HD, other recent studies have done so. In particular, we and others have noted that across both murine and human cellular HD models, astrocytic gene sets involved in both cytoskeletal maintenance and synaptic support are significantly impaired (41, 57). As such, the empiric structural observations of our study provide a supportive readout for the dysregulated transcriptional mechanisms identified in these parallel molecular studies. Thus, while morphological changes per se comprise only one aspect of the complex changes occurring within these perisynaptic environments, their attendant geometric readouts reflect disease-dependent transcriptional changes, and in doing so may reveal critical information regarding the stability and function of synapses in response to disease.

Querying the precise structural interactions between neuronal spines and glial processes has hitherto comprised a daunting technical challenge. Mouse protoplasmic astrocytes contain tens of thousands of spines within their individual domains, and human astrocytes may contain 1 to 2 orders of magnitude more synapses than their murine counterparts (58). The diffuse web-like nature of astrocytic processes within the neuropil complicates their study further, as the cells extend processes into the smallest reaches of the extracellular space. These astrocytic domains and their encompassed synapses are difficult to probe in detail using light-microscopy, and yet at the same time are difficult to associate with phenotype using serial EM. Overcoming these obstacles, we found that glial–synaptic interactions within these domains can indeed be defined, captured, imaged, and analyzed topologically at the nanometer scale, using the serial combination of lentiviral astrocytic tagging, rabies tagging of coupled neuronal pairs, multiphoton imaging with laser, and X-ray registration, all followed by correlated light and serial block-face EM, as we have outlined. Using this approach, we found significant structural abnormalities in the astrocytic engagement and investiture of striatal synapses in the R6/2 mouse model of HD. In particular, we noted a pattern of diminished engagement of mature synapses by perisynaptic astrocytes, which suggested the diminished sequestration of those synapses, by glia already deficient in K^+^ and glutamate uptake mechanisms. Such disease-associated synaptic pathology may result in the local spread of synaptic glutamate and K^+^ into adjacent synaptic fields, and thereby contribute to the high interstitial K^+^ and attendant hyperexcitability of the R6/2 striatum. More broadly though, the methodologic pipeline that we have outlined in this study allows for the topological investigation of glial–synaptic relationships within any parenchymal domain of interest, whose presynaptic and postsynaptic components are known and for which associated glial components may be prospectively tagged—whether they are of astrocytic, microglial, or progenitor phenotype, and whether of host or exogenous origin. As such, we expect this approach to be broadly useful in defining synaptic microstructure in neurodegenerative and neuropsychiatric disorders, providing nanometer-scale insight into the integrity of involved synapses in disease states and, going forward, of the structural plasticity of these synapses in response to therapeutic manipulation.

## Materials and Methods

### Animals.

All experiments were approved by the Animal Ethics Committee of the University of Copenhagen (Afdeling for Ekperimentel Medicin, AEM Project Plan P-20-054), as well as by the Institutional Animal Care and Use Committee of the University of Rochester. WT females with ovary transplants from R6/2^+^ (120 ± 5 CAG) donor mice were purchased from Jackson Laboratories (Bar Harbor, ME). The mice were genotyped following weaning, and double heterozygous mice were further analyzed to determine their CAG repeat number through PCR with primers encoding a product spanning the repeat region as previously described (59). All experiments included four to five mice/group, as indicated, with equal numbers of males and females.

### Induction of Fluorescence.

Rag1^−/−^ and Rag1^−/−^ × R6/2 mice were given two viral injections to induce EGFP and tRFP expression in MSNs and astrocytes, respectively. The mice were anesthetized with isofluorane flow, and Xylocaine was used as a local anesthetic (0.2 mg/mL), inserting the needle under the scalp and retracting the tip as the solution was delivered. At 10 wk of age, the mice received 500 nL bilateral injections of Lenti-GFAP-tRFP in the striatum at the following coordinates: AP +0.5 mm, ML ± 2.0 mm, and DV −3.4 mm from the bregma. After surgery, the mice were observed during waking and given the non-steroidal anti-inflammatory drug carprofen for pain management (1 mg/mL or 0.1 mL for a 20 g mouse). At 11 wk of age, the same operative procedures were followed, but this time with bilateral delivery of replication-inefficient rabies virus RV-dG-EGFP (generously provided by Dr. Ed Callaway) into the globus pallidus (coordinates: AP −0.3 mm, ML ± 1.7 mm, and DV −3.5 mm) (Fig. 1 *A* and *B*) (11).

One week later, the animals were transcardially perfused with 15 mL of Hanks’ basic salt solution (HBSS) (25 °C), followed by 20 to 25 mL of modified Karnovsky’s solution for fluorescence preservation in EM microscopy applications (2.5% glutaraldehyde, 2.0% paraformaldehyde (PFA) in 0.15 M cacodylate buffer, and 2 mM CaCl_2_, pH 7.2 to 7.4). The brains were then extracted and postfixed for 2 to 24 h. Following postfixation, the hemispheres were separated sagitally, and slices were cut at 500 to 700 µm thickness. The slices were then washed in cold phosphate-buffered saline (PBS) (pH 7.4) 3 × 5 min and immersed in sodium borohydrate (10 mg/mL) in PBS to quench glutaraldehyde autofluorescence for 30 min to 1 hr. The samples were then washed once more in cold PBS 3 × 5 min and transferred to glass slides.

### 2-Photon Imaging.

The samples were moisturized with one or two drops of ice-cold PBS and covered with Grace Bio-Labs Coverwell imaging chambers. This prevented any shifting in the position of the slice during imaging and further maintained the internal moisture from evaporating during the course of the 2-photon image acquisition. We used a ThorLabs Bergamo II microscope with a resonant scanner and a DeepSee Laser with tunable wavelength, using a 25X LWD water immersion objective from Nikon (NA 1.1). We then probed the striatum to find regions where MSNs expressing EGFP could be seen to interact with tRFP-expressing astrocytes. These tended to occur at the boundary of the striatal injection site, as the region surrounding the needle path contained too many labeled astrocyte domains to distinguish which astrocyte was servicing which dendrite.

Multiple ROIs were selected where appropriate, and these were scanned at several digital magnification settings, ranging from 1× to capture a general overview of the ROI up to 8× to record information relevant to individual branches. We used 900 to 950 nm excitation for the green channel and 1,000 to 1,050 nm to identify red (astrocytes); all image stacks were recorded at 1280p resolution with 0.3 to 1.0 µm z-steps, depending on the optical zoom and desired detail for that configuration (4× and 8× digital zoom stacks were obtained at 0.3 µm z-steps). Following both detailed and overview stack captures, regions were selected for the execution of the NIRB protocol (14). The laser was set to 900 to 950 nm, and power settings were adjusted to at least 200 mW at the back focal plane of the objective. Box scan settings were used with the resonant scanner, and small rectangular ablations were made in the vicinity of the ROI for multiple purposes. Larger ablations were made within 300 µm of the ROIs at lower zoom settings (1× or 2× digital zoom), while smaller ablations at higher digital zoom settings were made within 50 µm of the desired ROI dendritic branches (Movie S2). These were closely monitored during generation until the tissue displayed autofluorescence and the markings were clearly visible in transmitted light. The ROIs were once more scanned postbranding to record image stacks of the dendritic branches, astrocyte domains, and their relative orientation with respect to the induced ablations. The sagittal sections were removed from the imaging chambers, and ROIs were excised with a microscalpel under transmitted light. Samples were then immersed in 4% glutaraldehyde and stored for no more than 24 h before EM treatment.

### EM Sample Treatment.

The samples were prepared in accordance with the Ellisman’s lab protocol, designed to enhance the signal for backscatter electron detection of epoxy-embedded mammalian tissue at low accelerating voltages (60). The sample treatment was selected due to its focus on highlighting membrane contrast. All solutions were made with double distilled water (ddH_2_O) unless otherwise indicated, and all solutions were made fresh for each set of concurrent samples to be prepared. Samples were kept in ice-cold fixative (2.5% glutaraldehyde, 2.0% PFA, 2mM CaCl, and 0.15 M cacodylate buffer) for 2 to 3 h. Samples were rinsed in cold cacodylate buffer containing 2 mM CaCl for 3 × 5 min. Right before use, we mixed equal parts of 4% osmium tetroxide and 3% potassium ferrocyanide in 0.3M cacodylate buffer with 4 mM CaCl. The tissue was submerged in this solution for 1 h, ice cold. Samples were rinsed again 3 × 5 min with ddH_2_O at room temperature and incubated in thiocarbohydrazide (TCH, 0.22 mm Millipore filtered) solution for 20 min at room temperature, followed by 3 × 5min rinse with ddH_2_O. Following rinsing, we incubated with 2% osmium tetroxide in ddH_2_O for 30 min at RT. Samples were once more rinsed 3 × 5 min with ddH_2_O at RT and then incubated in 1% uranyl acetate at 4 °C overnight. Afterward, samples were rinsed 3 × 5 min with ddH_2_O at RT followed by en bloc lead aspartate staining for 30 min at 60 °C (0.02 lead nitrate in 0.03 M lead citrate, pH 5.5). Samples were washed once more 3 × 5 min with ddH_2_O and dehydrated in a series of grade ethanol concentrations (20%, 50%, 70%, 90%, 99%, and 100% anhydrous). Each immersion was done 2 × 7 min, and then, samples were placed in anhydrous ice-cold acetone for 10 min and transferred to RT acetone for 10 min thereafter. Infiltration with resin was done also with a graded series of Durcupan:acetone (25%, 50%, 75%, and 100%), each for 2 to 3 h at RT with the final step incubating overnight. The next day, samples were immersed in fresh 100% Durcupan (Sigma) for 6 h and embedded in resin, using flat molds to polymerize over 48 h at 60 °C in the oven.

### MicroCT Imaging (X-Ray).

We used a Versa520 microCT from Zeiss for the X-ray tomography of the embedded tissue blocks. The excess resin was trimmed as much as possible from the boundaries of the blocks, and the sample was positioned as close as possible to the X-ray source to achieve the highest possible resolution. The versa 520 utilizes a cone-shaped source, and the effective voxel size depends on the distance between the detector and the sample. The sample was glued to a holder and scanned twice at 40× magnification. The first scan was done at low voxel resolution to capture the entirety of the sample and pinpoint the location where the large 2-photon ablations could be clearly discerned. The second scan was done at the ROI at 0.6 µm voxel resolution to better resolve the location of the somas within the tissue block and properly overlay the fluorescence dataset using imaging software. The scans were done at 60 kV accelerating voltage and 2 s dwell time for low-resolution scans or 10 s dwell time for high-resolution scans of the ROI region.

### Correlation of X-Ray and 2-Photon Datasets.

Both X-ray datasets (low- and high-resolution settings) were loaded onto Avizo Amira (ThermoFisher) and aligned accordingly. Cell somas, 2-photon fiduciary ablations, and blood vessels were used to overlay the 2-photon datasets on the correct region of the X-ray datasets. Once the appropriate location was selected by cross-referencing using all the available X-ray and 2-photon datasets, the block was further trimmed down to just one column of tissue with sides roughly 300 to 350 µm in length (*SI Appendix*, Fig. S1*A*). This was necessary because the microtome blade of the SEM cannot accommodate for consistent surface cuts if the dimensions of the block face exceed 400 µm in length. Blood vessels, cell nuclei, and induced IR ablations were all used to identify depth, blade angle of attack, and xy coordinates of ROI before selecting the trimming region (*SI Appendix*, Fig. S1*B*).

### SBF-SEM Imaging.

In preparation for SBF-SEM, the trimmed sample was sputter coated with 10 nm of gold and inserted into the sample holder of the SBF SEM TeneoVS system from FEI (Thermo Fisher). The sample surface was polished at a blade speed of 400 mm/s and 100 nm thickness until at least 25% of the block surface was revealed. Because the process of SBF-SEM data acquisition is destructive, it is necessary to continuously trim the resin on the sample and estimate the distance to the forthcoming ROI beneath the surface (*SI Appendix*, Fig. S1*B*). The system was then pumped down to vacuum and allowed to stabilize overnight. We then captured a series of preliminary 20 to 30 slices at 50 nm thickness, preferably in regions containing vascular details (1.78 keV and 10 nA, 2048 by 1768 pixels, 3 µs pixel dwell time, and 1000 to 5000× magnification). ROI-specific data were acquired at 40-nm cuts. We used two energies for true 20 nm axial resolution (1.78 and 2.7 keV, 10 nA current, 2 µs dwell time) and corresponding image xy resolution of 6 to 7 nm pixel size and captured ~200 to 300 slices per block.

### Data Alignment and Segmentation.

The captured data were aligned and cross-referenced against the microCT scans and 2-photon data prior to segmentation. Once the acquired serial EM block was aligned and confirmed to contain the ROI of interacting astrocyte and MSN domains, the relevant dendritic branches were manually segmented with the use of Amira software (ThermoFisher Scientific; Movie S1). The dendritic spines studied were all components of astrocyte-engaged synapses. We verified that the investigated spines were apposed to presynaptic terminals with synaptic vesicles and that these established contact with postsynaptic spine structures displaying postsynaptic densities. To allow reliable quantification of astrocytic morphometrics at the synaptic periphery, we focused on the relationship of astrocytic processes to the PSD, as a more clearly defined reference point than the presynaptic terminal. Astrocytic processes surrounding these dendritic spines were then reconstructed locally. For well-defined ROIs containing dendritic spines engaged by astrocytic processes, the thin and mushroom spines were also identified and segmented. As a standard, we utilized the same color code for all EM datasets, where the dendritic spine was labeled green, astrocyte in blue, axon terminal in red, and PSD in yellow (Movie S3). These classes were defined according to the existence of clearly defined PSD structures and proximal astrocyte processes surrounding the synaptic cleft.

### Automated Structural Analysis from Geometry Derivation.

The automated analytical method used is based on a derivation of the n-dimensional Hausdorff measurements obtained from the interactions of an observed object with respect to a reference object (61, 62), in this case, to assess the quantitative information associated with the intersecting planes of the astrocyte process and the PSD (16). We measured the shape and position of the observed astrocyte process (X) with respect to the reference PSD object (Y) at increasing radii *r* from the PSD boundary. Iteratively, we dilate the PSD structure at some distance *r* and investigate the relationship between the PSD-based boundary and the observed object. Here, we measure the section of the astrocyte process that lies within distance *r* from the PSD, denoted Y, where the set of all points within this distance *r* that belong to the astrocyte is called the *r-parallel* set of Y, denoted Y^r^. Therefore, the coordinates corresponding to the astrocyte process (X) object contained within Y^r^, comprise the intersection of the PSD-derived surface and the astrocyte process at that distance *r*, denoted X ∩ Y^r^.

The interpretation of the intersecting PSD–astrocyte boundary is expanded in Fig. 3, with the corresponding equivalent measurement when the same interaction is observed in 3D parameters. Thus, for any X ∩ Y^r^ where r ≤ 1,000 nm from Y, we measured the volume and outer boundary surface area of the intersection boundary parameters of X at distance *r*. Derivation of Hausdorff measurements for intersecting boundaries yielded two additional geometric parameters (surface area parallel to reference object and length of intersection perimeter), but these metrics do not translate to intelligible biological analogues, whereas the surface area can be directly understood as the astrocyte membrane and the volume as the internal contents of the astrocyte process. All measurements were normalized for spherical parameters, e.g., volume increase at each r distance was normalized with regard to equivalent total volume of the dilated surface at the same radius, and both normalized and unnormalized results are reported (Fig. 4 and *SI Appendix*, Fig. S4). Data were reported for recorded results from an interval of radii representing close proximity to the PSD (25 to 175 nm from PSD).

## Data Analysis.

All statistical comparisons were made with the use of GraphPad Prism 8. Comparisons for nonequal variances, two-tailed *t* test, were used for Fig. 2 data. Quantitative results are shown as mean ± SEM, and statistical significance was accepted at *P* < 0.05. For Fig. 4, in order to estimate the statistical significance of the difference between the measurement curves for the WT and R6/2 groups, we implemented the Monte Carlo permutation test. The distance t’ between the WT and R6/2 mean curves was first measured by the absolute area under the curve. We then collected the graphs from both groups and randomly sampled two new groups from the full collection of curves *n* times and measured the distance t_1_, […], t_n_. The fraction of t_1_, […], t_n_ < t’ determines the *P* value in a stochastic manner (16).

## Supplementary Material

Appendix 01 (PDF)

Dataset S01 (XLSX)

Movie S1. **Correlative microscopy, 2 photon and X-ray imaging for ROI definition** *Related to Figure 1* X-ray datasets of samples were used as a main navigation tool, whereby the higher resolution 2 photon datasets from preceding captures are overlayed onto both fiduciary marks and the microvasculature, to determine the correct x-y-z coordinates of the prospectively defined neuron-astrocyte region-of-interest (ROI), for subsequent serial SEM imaging. Note that the reconstructed MSN details the difference between the 2 photon-segmented and serial EM datasets.

Movie S2. **Relocalization of EM stack, after X-ray and 2 photon correlated light EM (CLEM)** *Related to Figure S1* This video presents the EM environment with iteratively higher magnification scans. Multiple EM stack correlations are necessary, since serial block-face SEM is a destructive imaging process, and there is only one opportunity to capture each desired ROI stack. The reconstructed medium spiny neuron branch shown here is the same as that in video 1, with dendritic spines added to show the neuron-glia environments selected for analysis.

Movie S3. **Reconstructed neuropil volume of striatum and perisynaptic astrocyte** *Related to Figure 2* This selected subvolume of striatum shows the density of the segmented environment. The camera path is focused on a single synapse, and shows a prototypic example of astrocytic coverage of the perisynaptic region. Multiple views reveal the complex association of astrocytes with both the pre- and post-synaptic components of mature, mushroom-like synapses, the proximal elements of which were used for the analysis strategy described in Figure 2 (see segment with opaque and transparent overlay of astrocyte, at 00:22:00).

Movie S4. **Identification and characterization of perisynaptic morphologies** *Related to Figures S2 and S3* We observed a variety of configurations of astrocytic engagement with MSN spines, with a spectrum of astrocytic infiltration patterns observed at each analyzed level of dendritic spine maturity. These examples focus on the prototypic engagement of mature spines in WT mice, in which tight astrocytic sequestration of the perisynaptic space was typically observed.

## Data Availability

Imaging files, data, and code data have been deposited in the Goldman lab database for this paper: http://qim.dk/portfolio-items/relational-shape-measure/?portfolioCats=16 (63); https://lab.compute.dtu.dk/QIM/tools/relationalshapemeasure (64). All study data are included in the article and/or supporting information.

## References

[1] M. E. Ehrlich, Huntington’s disease and the striatal medium spiny neuron: Cell-autonomous and non-cell-autonomous mechanisms of disease. Neurotherapeutics **9**, 270–284 (2012). 22441874 10.1007/s13311-012-0112-2 PMC3337013

[2] N. Bazargani, D. Attwell, Astrocyte calcium signaling: The third wave. Nat. Neurosci. **19**, 182–189 (2016). 26814587 10.1038/nn.4201

[3] N. J. Allen, C. Eroglu, Cell biology of astrocyte-synapse interactions. Neuron **96**, 697–708 (2017). 29096081 10.1016/j.neuron.2017.09.056 PMC5687890

[4] K. Runge, C. Cardoso, A. de Chevigny, Dendritic spine plasticity: Function and mechanisms. Front. Synaptic Neurosci. **12**, 36 (2020). 32982715 10.3389/fnsyn.2020.00036 PMC7484486

[5] S. Basu , Quantitative 3-D morphometric analysis of individual dendritic spines. Sci. Rep. **8**, 3545 (2018). 29476060 10.1038/s41598-018-21753-8 PMC5825014

[6] E. Bertling, A. Ludwig, M. Koskinen, P. Hotulainen, Methods for three-dimensional analysis of dendritic spine dynamics. Methods Enzymol. **506**, 391–406 (2012). 22341234 10.1016/B978-0-12-391856-7.00043-3

[7] C. G. Langhammer , Automated sholl analysis of digitized neuronal morphology at multiple scales: Whole cell sholl analysis versus sholl analysis of arbor subregions. Cytometry A **77**, 1160–1168 (2010). 20687200 10.1002/cyto.a.20954 PMC4619108

[8] A. Rodriguez, D. B. Ehlenberger, D. L. Dickstein, P. R. Hof, S. L. Wearne, Automated three-dimensional detection and shape classification of dendritic spines from fluorescence microscopy images. PLoS One **3**, e1997 (2008). 18431482 10.1371/journal.pone.0001997 PMC2292261

[9] J. C. Octeau , An optical neuron-astrocyte proximity assay at synaptic distance scales. Neuron **98**, 49–66.e49 (2018). 29621490 10.1016/j.neuron.2018.03.003 PMC5916847

[10] S. J. Miller, J. D. Rothstein, Astroglia in thick tissue with super resolution and cellular reconstruction. PLoS One **11**, e0160391 (2016). 27494718 10.1371/journal.pone.0160391 PMC4975496

[11] I. R. Wickersham, S. Finke, K. K. Conzelmann, E. M. Callaway, Retrograde neuronal tracing with a deletion-mutant rabies virus. Nat. Methods **4**, 47–49 (2007). 17179932 10.1038/NMETH999 PMC2755236

[12] G. J. Klapstein , Electrophysiological and morphological changes in striatal spiny neurons in R6/2 huntington’s disease transgenic mice. J. Neurophysiol. **86**, 2667–2677 (2001). 11731527 10.1152/jn.2001.86.6.2667

[13] M. A. Karreman , Find your way with X-Ray: Using microCT to correlate in vivo imaging with 3D electron microscopy. Methods Cell Biol. **140**, 277–301 (2017). 28528637 10.1016/bs.mcb.2017.03.006

[14] D. Bishop , Near-infrared branding efficiently correlates light and electron microscopy. Nat. Methods **8**, 568–570 (2011). 21642966 10.1038/nmeth.1622

[15] P. M. Dixon, “R ipley’s K function” in StatsRef: Statistics Reference Online (Wiley, 2014).

[16] H. J. T. Stephensen, C. B. Villanueva, A. Svane, S. A. Goldman, J. Sporring, Measuring shape relations using r-parallel sets. J Math Imaging Vis **63**, 1069–1083 (2021).

[17] I. Patrushev, N. Gavrilov, V. Turlapov, A. Semyanov, Subcellular location of astrocytic calcium stores favors extrasynaptic neuron-astrocyte communication. Cell Calcium **54**, 343–349 (2013). 24035346 10.1016/j.ceca.2013.08.003

[18] N. Gavrilov , Astrocytic coverage of dendritic spines, dendritic shafts, and axonal boutons in hippocampal neuropil. Front. Cell Neurosci. **12**, 248 (2018). 30174590 10.3389/fncel.2018.00248 PMC6108058

[19] M. R. Witcher, S. A. Kirov, K. M. Harris, Plasticity of perisynaptic astroglia during synaptogenesis in the mature rat hippocampus. Glia **55**, 13–23 (2007). 17001633 10.1002/glia.20415

[20] M. Haber, L. Zhou, K. K. Murai, Cooperative astrocyte and dendritic spine dynamics at hippocampal excitatory synapses. J. Neurosci. **26**, 8881–8891 (2006). 16943543 10.1523/JNEUROSCI.1302-06.2006 PMC6675342

[21] I. Lushnikova, G. Skibo, D. Muller, I. Nikonenko, Synaptic potentiation induces increased glial coverage of excitatory synapses in CA1 hippocampus. Hippocampus **19**, 753–762 (2009). 19156853 10.1002/hipo.20551

[22] N. Medvedev , Glia selectively approach synapses on thin dendritic spines. Philos. Trans. R Soc. Lond. B Biol. Sci. **369**, 20140047 (2014). 25225105 10.1098/rstb.2014.0047 PMC4173297

[23] A. Dunaevsky, A. Tashiro, A. Majewska, C. Mason, R. Yuste, Developmental regulation of spine motility in the mammalian central nervous system. Proc. Natl. Acad. Sci. U.S.A. **96**, 13438–13443 (1999). 10557339 10.1073/pnas.96.23.13438 PMC23966

[24] R. Yuste, T. Bonhoeffer, Morphological changes in dendritic spines associated with long-term synaptic plasticity. Annu. Rev. Neurosci. **24**, 1071–1089 (2001). 11520928 10.1146/annurev.neuro.24.1.1071

[25] M. E. Dailey, S. J. Smith, The dynamics of dendritic structure in developing hippocampal slices. J. Neurosci. **16**, 2983–2994 (1996). 8622128 10.1523/JNEUROSCI.16-09-02983.1996 PMC6579052

[26] J. Noguchi, M. Matsuzaki, G. C. Ellis-Davies, H. Kasai, Spine-neck geometry determines NMDA receptor-dependent Ca2+ signaling in dendrites Neuron **46**, 609–622 (2005). 15944129 10.1016/j.neuron.2005.03.015 PMC4151245

[27] B. Engelhardt, R. M. Ransohoff, The ins and outs of T-lymphocyte trafficking to the CNS: Anatomical sites and molecular mechanisms. Trends Immunol. **26**, 485–495 (2005). 16039904 10.1016/j.it.2005.07.004

[28] J. Smolders , Characteristics of differentiated CD8(+) and CD4 (+) T cells present in the human brain. Acta Neuropathol. **126**, 525–535 (2013). 23880787 10.1007/s00401-013-1155-0

[29] W. F. Hickey, Leukocyte traffic in the central nervous system: The participants and their roles. Semin. Immunol. **11**, 125–137 (1999). 10329499 10.1006/smim.1999.0168

[30] M. R. von Essen , Early intrathecal T helper 17.1 cell activity in huntington disease. Ann. Neurol. **87**, 246–255 (2020). 31725947 10.1002/ana.25647

[31] P. O. McGowan, T. A. Hope, W. H. Meck, G. Kelsoe, C. L. Williams, Impaired social recognition memory in recombination activating gene 1-deficient mice. Brain Res. **1383**, 187–195 (2011). 21354115 10.1016/j.brainres.2011.02.054 PMC3436067

[32] L. Rattazzi, A. Cariboni, R. Poojara, Y. Shoenfeld, F. D’Acquisto, Impaired sense of smell and altered olfactory system in RAG-1(-∕-) immunodeficient mice. Front. Neurosci. **9**, 318 (2015). 26441494 10.3389/fnins.2015.00318 PMC4563081

[33] L. Rattazzi , CD4(+) but not CD8(+) T cells revert the impaired emotional behavior of immunocompromised RAG-1-deficient mice. Transl. Psychiatry **3**, e280 (2013). 23838891 10.1038/tp.2013.54 PMC3731786

[34] J. Cushman, J. Lo, Z. Huang, C. Wasserfall, J. M. Petitto, Neurobehavioral changes resulting from recombinase activation gene 1 deletion. Clin. Diagn. Lab. Immunol. **10**, 13–18 (2003). 12522033 10.1128/CDLI.10.1.13-18.2003 PMC145286

[35] G. Laumet, J. D. Edralin, R. Dantzer, C. J. Heijnen, A. Kavelaars, CD3(+) T cells are critical for the resolution of comorbid inflammatory pain and depression-like behavior. Neurobiol. Pain **7**, 100043 (2020). 32510006 10.1016/j.ynpai.2020.100043 PMC7264986

[36] X. Tong , Astrocyte Kir4.1 ion channel deficits contribute to neuronal dysfunction in Huntington’s disease model mice. Nat. Neurosci. **17**, 694–703 (2014). 24686787 10.1038/nn.3691 PMC4064471

[37] M. Osipovitch , Human ESC-derived chimeric mouse models of Huntington’s disease reveal cell-intrinsic defects in glial progenitor cell differentiation. Cell Stem Cell **24**, 107–122.e107 (2019). 30554964 10.1016/j.stem.2018.11.010 PMC6700734

[38] A. Benraiss , Human glia can both induce and rescue aspects of disease phenotype in Huntington disease. Nat. Commun. **7**, 11758 (2016). 27273432 10.1038/ncomms11758 PMC4899632

[39] C. Cepeda, D. M. Cummings, V. M. Andre, S. M. Holley, M. S. Levine, Genetic mouse models of Huntington’s disease: Focus on electrophysiological mechanisms. ASN Neuro **2**, e00033 (2010). 20396376 10.1042/AN20090058 PMC2850512

[40] M. Valenza , Disruption of astrocyte-neuron cholesterol cross talk affects neuronal function in Huntington’s disease. Cell Death Differ **22**, 690–702 (2015). 25301063 10.1038/cdd.2014.162 PMC4356339

[41] B. Diaz-Castro, M. R. Gangwani, X. Yu, G. Coppola, B. S. Khakh, Astrocyte molecular signatures in Huntington’s disease. Sci. Transl. Med. **11**, eaaw8546 (2019). 31619545 10.1126/scitranslmed.aaw8546

[42] J. Jin , Early white matter abnormalities, progressive brain pathology and motor deficits in a novel knock-in mouse model of Huntington’s disease. Hum. Mol. Genet **24**, 2508–2527 (2015). 25609071 10.1093/hmg/ddv016 PMC4383863

[43] R. T. Teo , Structural and molecular myelination deficits occur prior to neuronal loss in the YAC128 and BACHD models of Huntington disease. Hum. Mol. Genet. **25**, 2621–2632 (2016). 27126634 10.1093/hmg/ddw122 PMC5181633

[44] A. M. Estrada-Sanchez, G. V. Rebec, Corticostriatal dysfunction and glutamate transporter 1 (GLT1) in Huntington’s disease: Interactions between neurons and astrocytes. Basal Ganglia **2**, 57–66 (2012). 22905336 10.1016/j.baga.2012.04.029 PMC3418680

[45] J. Y. Shin , Expression of mutant huntingtin in glial cells contributes to neuronal excitotoxicity. J. Cell Biol. **171**, 1001–1012 (2005). 16365166 10.1083/jcb.200508072 PMC2171327

[46] M. Faideau , In vivo expression of polyglutamine-expanded huntingtin by mouse striatal astrocytes impairs glutamate transport: A correlation with Huntington’s disease subjects. Hum. Mol. Genetics **19**, 3053–3067 (2010). 10.1093/hmg/ddq212 PMC2901144 20494921

[47] Y. Bernardinelli, D. Muller, I. Nikonenko, Astrocyte-synapse structural plasticity. Neural. Plast **2014**, 232105 (2014). 24511394 10.1155/2014/232105 PMC3910461

[48] Y. Bernardinelli , Activity-dependent structural plasticity of perisynaptic astrocytic domains promotes excitatory synapse stability. Curr. Biol. **24**, 1679–1688 (2014). 25042585 10.1016/j.cub.2014.06.025

[49] M. Matsuzaki , Dendritic spine geometry is critical for AMPA receptor expression in hippocampal CA1 pyramidal neurons. Nat. Neurosci. **4**, 1086–1092 (2001). 11687814 10.1038/nn736 PMC4229049

[50] O. Ganeshina, R. W. Berry, R. S. Petralia, D. A. Nicholson, Y. Geinisman, Synapses with a segmented, completely partitioned postsynaptic density express more AMPA receptors than other axospinous synaptic junctions. Neuroscience **125**, 615–623 (2004). 15099675 10.1016/j.neuroscience.2004.02.025

[51] O. Ganeshina, R. W. Berry, R. S. Petralia, D. A. Nicholson, Y. Geinisman, Differences in the expression of AMPA and NMDA receptors between axospinous perforated and nonperforated synapses are related to the configuration and size of postsynaptic densities. J. Comp. Neurol. **468**, 86–95 (2004). 14648692 10.1002/cne.10950

[52] M. C. Ashby, S. R. Maier, A. Nishimune, J. M. Henley, Lateral diffusion drives constitutive exchange of AMPA receptors at dendritic spines and is regulated by spine morphology. J. Neurosci. **26**, 7046–7055 (2006). 16807334 10.1523/JNEUROSCI.1235-06.2006 PMC6673929

[53] E. A. Nimchinsky, R. Yasuda, T. G. Oertner, K. Svoboda, The number of glutamate receptors opened by synaptic stimulation in single hippocampal spines. J. Neurosci. **24**, 2054–2064 (2004). 14985448 10.1523/JNEUROSCI.5066-03.2004 PMC6730404

[54] R. P. Murmu, W. Li, A. Holtmaat, J. Y. Li, Dendritic spine instability leads to progressive neocortical spine loss in a mouse model of Huntington’s disease. J. Neurosci. **33**, 12997–13009 (2013). 23926255 10.1523/JNEUROSCI.5284-12.2013 PMC6619731

[55] S. J. Bulley, C. J. Drew, A. J. Morton, Direct visualisation of abnormal dendritic spine morphology in the hippocampus of the R6/2 transgenic mouse model of Huntington’s Disease. J. Huntingtons Dis. **1**, 267–273 (2012). 25063335 10.3233/JHD-120024

[56] A. Perez-Alvarez, M. Navarrete, A. Covelo, E. D. Martin, A. Araque, Structural and functional plasticity of astrocyte processes and dendritic spine interactions. J. Neurosci. **34**, 12738–12744 (2014). 25232111 10.1523/JNEUROSCI.2401-14.2014 PMC6705321

[57] A. Benraiss , Cell-intrinsic glial pathology is conserved across human and murine models of Huntington’s disease. Cell Rep. **36**, 109308 (2021). 34233199 10.1016/j.celrep.2021.109308

[58] N. A. Oberheim , Uniquely hominid features of adult human astrocytes. J. Neurosci. **29**, 3276–3287 (2009). 19279265 10.1523/JNEUROSCI.4707-08.2009 PMC2819812

[59] A. Benraiss , Sustained mobilization of endogenous neural progenitors delays disease progression in a transgenic model of Huntington’s disease. Cell Stem Cell **12**, 787–799 (2013). 23746982 10.1016/j.stem.2013.04.014 PMC4051319

[60] T. J. Deerinck , High-performance serial block-face SEM of nonconductive biological samples enabled by focal gas injection-based charge compensation. J. Microsc. **270**, 142–149 (2018). 29194648 10.1111/jmi.12667 PMC5910240

[61] D. G. Sim, R. H. Park, Two-dimensional object alignment based on the robust oriented Hausdorff similarity measure. IEEE Trans. Image Process **10**, 475–483 (2001). 18249637 10.1109/83.908541

[62] H. Federer, Hausdorff measure and lebesgue area. Proc. Natl. Acad. Sci. U.S.A. **37**, 90–94 (1951). 16578362 10.1073/pnas.37.2.90 PMC1063310

[63] H. J. T. Stephensen, A. M. Svane, J. Sporring, Center for Quantification of Imaging Data from MAX IV, Software Tools, Relational Shape Measure. QIM software tools repository. http://qim.dk/portfolio-items/relational-shape-measure/?portfolioCats=16. Deposited 19 December 2021.

[64] H. J. T. Stephensen, A. M. Svane, J. Sporring, Relational Shape Measure. DTU Compute GitLab Enterprise. https://lab.compute.dtu.dk/QIM/tools/relationalshapemeasure. Deposited 19 December 2021.

